# Few-layer graphene induces both primary and secondary genotoxicity in epithelial barrier models in vitro

**DOI:** 10.1186/s12951-021-00769-9

**Published:** 2021-01-19

**Authors:** Michael J. Burgum, Martin J. D. Clift, Stephen J. Evans, Nicole Hondow, Afshin Tarat, Gareth J. Jenkins, Shareen H. Doak

**Affiliations:** 1 Institute of Life Science, Swansea University Medical School, Swansea University, Singleton Park, Swansea, SA2 8PP Wales UK; 2 School of Chemical and Process Engineering, University of Leeds, Leeds, LS2 9JT UK; 3 Perpetuus Carbon Technologies, Unit B1, Olympus Court, Millstream Way, Llansamlet, Swansea Vale, SA70AQ UK

## Abstract

**Background:**

Toxicological evaluation of engineered nanomaterials (ENMs) is essential for occupational health and safety, particularly where bulk manufactured ENMs such as few-layer graphene (FLG) are concerned. Additionally, there is a necessity to develop advanced in vitro models when testing ENMs to provide a physiologically relevant alternative to invasive animal experimentation. The aim of this study was to determine the genotoxicity of non-functionalised (neutral), amine- and carboxyl-functionalised FLG upon both human-transformed type-I (TT1) alveolar epithelial cell monocultures, as well as co-cultures of TT1 and differentiated THP-1 monocytes (d.THP-1 (macrophages)).

**Results:**

In monocultures, TT1 and d.THP-1 macrophages showed a statistically significant (*p* < 0.05) cytotoxic response with each ENM following 24-h exposures. Monoculture genotoxicity measured by the in vitro cytokinesis blocked micronucleus (CBMN) assay revealed significant (*p* < 0.05) micronuclei induction at 8 µg/ml for amine- and carboxyl-FLG. Transmission electron microscopy (TEM) revealed ENMs were internalised by TT1 cells within membrane-bound vesicles. In the co-cultures, ENMs induced genotoxicity in the absence of cytotoxic effects. Co-cultures pre-exposed to 1.5 mM N-acetylcysteine (NAC), showed baseline levels of micronuclei induction, indicating that the genotoxicity observed was driven by oxidative stress.

**Conclusions:**

Therefore, FLG genotoxicity when examined in monocultures, results in primary-indirect DNA damage; whereas co-cultured cells reveal secondary mechanisms of DNA damage.
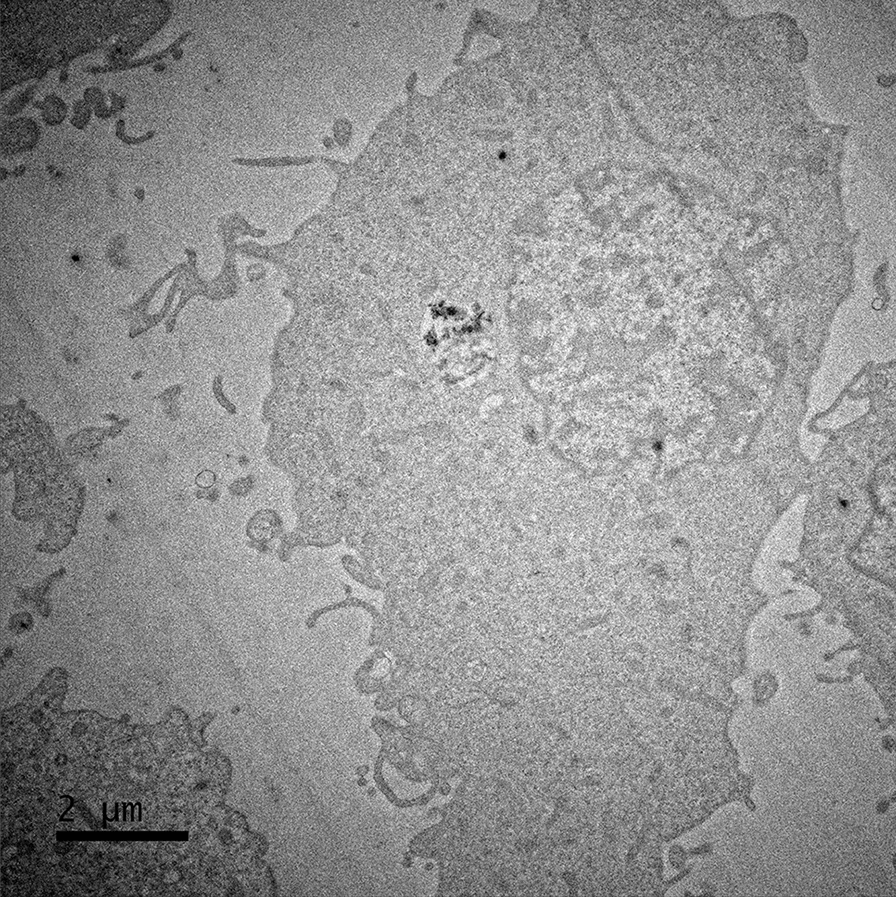

## Introduction

ENMs have unique physico-chemical features which provide the basis for novel applications in medicine, engineering and other such disciplines [26]. However, these unique physico-chemical features (size, shape, surface area, reactivity) cause concern as to their impact upon human health [15]. The graphene family of nanomaterials (GFNs) have received much attention due to their near-2-dimensional (2D) morphology and physico-chemical characteristics granting their use in energy storage and nanocomposites [10]. Despite the plethora of applications, there are still concerns as to the occupational and consumer exposure risks associated with graphene-based ENMs [1]. As shown in the work by Burgum and colleagues, FLG which can be bulk manufactured, can pose a risk to lung cells with DNA damage being related to the specific surface characterisation of the materials.

ENMs have in some cases, been reported to induce damage DNA which could, in turn, promote carcinogenesis [18, 21, 25]. Evaluation of the mechanisms underlying ENM DNA damage in vitro can be accomplished utilising monocultured cells to detect primary (indirect) genotoxicity or advanced co-culture models, which allow detection of secondary genotoxic mechanisms [8]. Primary genotoxic mechanisms are sub-classified into primary-direct and -indirect genotoxicity. Primary-direct genotoxicity is associated with the exogenous agent inducing DNA damage through physical interaction with DNA molecule itself. For instance, graphene oxide (GO) has been shown to intercalate into DNA and when combined with copper ions, inducing DNA scission [22]. Primary-indirect mechanisms represent those where the exogenous agent damages key molecules involved in DNA replication, for example, DNA repair enzymes, replication machinery, cell cycle proteins or spindle formation. Primary-indirect genotoxicity can also be attributed to mitochondrial damage, antioxidant depletion and generation of reactive oxygen/nitrogen species (ROS/RNS [1, 4, 9]. These indirect pathways for damage ultimately contribute to DNA lesions, double strand breaks, aneuploidy and oxidative DNA damage. Secondary genotoxicity is typically observed in vivo and is the result of DNA damage induced through a (sub)-chronic immune response, involving immune cell activation and recruitment, heightened inflammatory activity and subsequent oxidative stress, promoting genotoxicity in the surrounding epithelial cells [7]. Given both primary and secondary genotoxicity are important mechanisms for ENM induced DNA damage, it is important that they are both evaluated to develop a comprehensive understanding of a materials’ genotoxic potential.

Respiratory models are common in nanotoxicology due to the primary route of ENM exposure being inhalation, especially in an occupational environment [2]. A graphene nanoplatelet with a lateral diameter of 20 µm was theorised by Schinwald and colleagues to possess an aerodynamic diameter of  < 2.5 µm, representing a size fraction of particulate matter that is capable of reaching the alveolar region [24]. To the authors knowledge, little is known about the potential of FLG materials to elicit genotoxicity in advanced in vitro models of the lung. Burgum et al. investigated the genotoxic effects that FLG materials functionalised with no specific groups, amine, or carboxyl groups had on bronchial epithelial (16HBE14o^−^) cells [1]. Each FLG proved capable of eliciting significant oxidative and mitochondrial stress however only the carboxyl-FLG was shown to be non-genotoxic at concentrations of 100 µg/ml. The authors concluded this may be due to potential oxidative radical-scavenging properties of the carboxyl groups. It is unclear however if the same effects could be proved using alveolar models and furthermore, co-culture models. These models could highlight secondary mechanisms of FLG toxicity which can go unseen using in vitro monocultures which, constitute the bulk of the literature surrounding FLG genotoxicity. The current state of the literature pertaining to FLG genotoxicity is sparse, with no known studies conducted involving lung co-cultures to model FLG occupational hazard. The biological response of bone marrow-derived macrophages from mice were recently exposed to FLG at 3, 10, 30 and 100 µg/ml for 24 h in the study by Malanagahalli et al. FLG uptake by the macrophages was reported and confirmed by TEM, the authors reported no significant cytotoxic effects (measure with flow cytometry), as well as no significant rise in (pro)-inflammatory mediators [20]. The authors did conclude that whilst the results appear promising, more evidence is required to elucidate other immunological pathways and investigate the mechanistic effects of FLG. The impact of GO has been investigated on monocultures of J774.A1 macrophages by Ma and colleagues in 2015. In their work, GO of varying lateral sizes (350 nm-1300 nm) were exposed to macrophages to determine the level of activation, and (pro)-inflammatory response. The authors reported large diameter GO showed greater potential for plasma membrane interaction and activation of (pro)-inflammatory mediators IL-6 and TNF-α. M1 macrophages showed significant secretion of IL-1β. The authors noted that at 20 µg/ml exposures over 24 h exaggerated the expression of mRNA of IL-1β in human macrophages [19]. Evidently, there seems to be gaps in the literature surrounding the role of FLG toxicity, in lung models and the underlying mechanisms which have yet to be identified. Further, there doesn’t appear to be any literature which addresses FLG toxicity in relevant co-culture models which could reveal potentially adverse secondary mechanisms of toxicity otherwise missed in standard monoculture testing.

The present study aimed to assess the genotoxic potential of FLG in monocultured TT1 cells and a co-culture of TT1/d.THP-1 cells to evaluate both primary and secondary genotoxic mechanisms. It is important to note TT1 cells were chosen as they represent ideal models of alveolar barrier function. TT1 cells form tight junctions when cultured in vitro and provide a robust alveolar type-I model of the lung where the majority of the ENMs used in the present study would deposit. Macrophages were selected for being professional phagocytes, thus representing a good model of the alveolar region. Additionally, this is to the authors knowledge the first study to investigate FLG impact upon an alveolar co-culture. Understanding the mechanisms contributing to the induction of DNA damage was also explored, focusing on the role of oxidative stress and (pro)-inflammatory response. It was hypothesised that in monoculture exposures, the physico-chemical features of the graphene would highlight differences between materials based upon the biological response.

## Materials and methods

### Preparation of ENMs

Neutral-, amine-, and carboxyl-FLG were manufactured via dielectric barrier discharge of mined graphite by Perpetuus Carbon Technologies (PCT, UK). CB particles were sourced from (FLAMMRUSS 101, Lamp Black #8,235,102) Evonik Degussa Inorganic Materials, Frankfurt. All ENMs were supplied as powder and were suspended at a stock concentration of 10 mg/ml in double distilled water, which were then titrated to final concentrations with cell culture medium. Prior to exposures ENMs were sonicated in a 90 W Ultrasonic Bath (Fisher Scientific #FB15046) for 20 min at 37 °C to encourage destabilisation of agglomerate material.

### ENM characterisation

Each test ENM had previously been characterised by Burgum et al. [1]. Briefly, neutral-FLG, amine-FLG, carboxyl-FLG (as well as, to a lesser extent, CB particles) particle and agglomerate sizes were investigated with several techniques including,plunge-freeze scanning electron microscopy (SEM), dynamic light scattering (DLS), atomic force microscopy (AFM) and Raman spectroscopy. The agglomerate analysis suggested a difference in size between the particle types when suspended in cell culture media with the additional surface groups of amine-FLG and carboxyl-FLG increasing the average diameter by ~ 300 and ~ 400 nm respectively. The addition of surface groups reduced the average thickness of FLG layer number when measured by AFM, the greatest effect produced by carboxyl groups which lowered the calculated layer number from 50 to 4 atomic layers of graphene.

### Cell culture

The TT1 cell line was kindly donated by Professor Terry Tetley of Imperial College London, UK. This alveolar cell line had been previously transformed, immortalised and characterised by Kemp and colleagues as a model for ATI cells [16]. TT1 cells were thawed from liquid nitrogen and transferred to T25 flasks to initiate proliferation for 48 h or until reaching 70–80% confluency before sub-culturing into a T75. TT1 cells were cultured with DCCM-1 Media (Geneflow Ltd, UK) supplemented with 1% Penicillin/Streptomycin/L-Glutamine (Sigma, UK) and 10% New-born Calf Serum (NCS) (Sigma, UK). Sub-culturing of TT1 cells began with removing existing media and discarding, washing the adherent cells twice with pre-warmed PBS before adding 4 ml of thawed Trypsin–EDTA and incubated flat at 37 °C for 5 min to disassociate. Once cells detached, complete media was added (6 ml) to de-activate the Trypsin–EDTA solution and the cells transferred to 15 ml Falcon tubes to be centrifuged at 340*g* for 10 min. The resulting supernatant was discarded, cells re-suspended and seeded at 1 × 10^5^ and returned to the incubator at 37 °C. When seeding TT1 cells for toxicological assays, cells were counted by re-suspending and taking 10 µl and adding to 90 µl of fresh media then pipetting 10 µl into a Neubauer chamber. Cells in the four corners of the chamber were counted, averaged and multiplied by 10^4^ for the number of cells per ml. TT1 cells were sub-cultured every 3 days, reaching 90–100% confluency in that time. TT1 cells were not used in toxicological assays once exceeding 20 passages. THP-1 cells and differentiation were sourced and cultured as described by Evans and colleagues [9]. THP-1 monocytes were routinely sub-cultured and maintained at concentrations between 5 and 8 × 10^5^ cells/ml to avoid clumping and maintain a healthy population of cells.

### THP-1 differentiation

To differentiate, THP-1 cells were centrifuged at 130*g* for 5 min at room temperature, following this the cells were resuspended in a 15 ml Falcon tube at a concentration of 5 × 10^5^ cells/ml with 50 nM of phorbol-12-myristate-13-acetate (PMA). This suspension was transferred to a T75 flask and incubated at 37 °C for 24 h. Cells were checked for morphology via light microscopy; differentiation was evident when cells did not detach from flask over gentle agitation. On the day of seeding co-cultures, d.THP-1 macrophages were resuspended at 1 × 10^5^ cells/ml in DCCM-1 media.

### Construction of co-culture model

The alveolar lung epithelial co-culture model was comprised of TT1 alveolar cells (seeded at 1 × 10^6^ cells/ml) and d.THP-1 macrophages (1 × 10^5^ cells/ml per 6-well insert). These co-cultures were constructed upon the apical side of PET track-etched 4.2 cm^2^ transwell inserts (3 µm pores) supported by a 6-well companion plate (Corning, Germany). TT1 cells were seeded on day one and left to proliferate for one week. At day five, THP-1 cells were differentiated into macrophages using PMA and left for 24 h. On day six, d.THP-1 macrophages were lifted with Acutase and transferred to the TT1 monolayer and left to adhere for one day. The completed co-culture was then ready to be used on day seven, this was confirmed with confocal laser scanning microscopy (LSM (images not shown)).

### Confirmation of cellular interaction/entry using Transmission Electron Microscopy (TEM)

ENM cellular uptake was confirmed by TEM imaging. TT1 cells, the primary focus of this study were exposed to ENM were fixed, embedded, sectioned and imaged as previously described [27]. The analysis was performed with a FEI Titan3 Themis G2 operating at 300 kV fitted with 4 EDX silicon drift detectors, and a Gatan One-View CCD. EDX spectroscopy and mapping to identify potential heavy metal contamination was undertaken using Bruker Esprit v1.9 software.

### TEM analysis of FLG lattice planes

In conjunction with TEM uptake imaging, confirmation of graphitic materials was confirmed through fast Fourier transform (FFT) using ImageJ. To begin analysis, firstly an intracellular vesicle containing an FLG crystal oriented on its z-axis was located, the image was captured and saved for ImageJ analysis. Using ImageJ software, the captured image was uploaded, the scale bar was set, and the lattice plane focused upon and analysed with FFT to produce an image (Additional file 1: Fig. S1) which reveals the interlayer spacing of FLG.

### Cytotoxicity by relative population doubling (RPD) & genotoxicity.

TT1 cells were seeded at 1.0 × 10^5^ cells/ml and allowed to adhere for 24 h after which the cells were then treated with ENMs for 24 h. Cell culture media was added as the negative control, mitomycin-C (MMC) at 0.01 μg/ml was used as the positive control, the assay was performed as previously detailed by [9]. All experiments were performed in triplicate (*N* = 3) and 2000 binucleate (BN) cells per replicate were scored for the presence of micronuclei per concentration (6000 BN cells in total).

### d.THP-1 cytotoxicity

Cytotoxicity of d.THP-1 macrophages (seeded at 1 × 10^5^ cells/ml) was assessed by trypan blue exclusion following 24-h exposure to ENMs as described by Evans and colleagues [9]. Following exposure cells were lifted gently with Acutase and live cells scored with a haemocytometer (1:5 dilution). This was performed in triplicate.

### (Pro)-inflammatory response

Supernatant from the CBMN assay was harvested following 24-h exposure to ENMs and analysed using an IL-6 & IL-8 ELISA (DuoSet ELISA; R&D Systems Europe). ELISA’s were performed in triplicate following the manufactures instructions. The optical density (OD) was recorded at a wavelength set to 450 nm with an Omega Multimode microplate reader (BMG LABTECH Ltd, UK). This was performed in triplicate.

### Mitochondrial stress

TT1 cells were seeded at 1 × 10^5^ cells/ml (100 µl/well) into XFe24 tissue culture plates (Seahorse Bioscience). The following day, 150 µl of growth media was added and exposed to ENMs for 24 h. The assay was performed as described in the work by Burgum et al. [1]. Data was normalised through quantification of cellular protein (DC Assay (Bio-Rad, UK)) and a series of BSA (Bio-Rad, UK) prepared using RIPA buffer (Thermo Scientific, UK). This was performed in triplicate.

### Co-culture in vitro CBMN assay

The in vitro CBMN assay was adapted for use with advanced co-culture models and performed as described in detail by Evans and colleagues [9]. As outlined by Doak and Elespuru, specific adaptations were made to the standard version of the assay which incorporates the handling of ENMs which differs from the use of chemical compounds [5, 7]. The authors followed OECD TG487 to evaluate cytotoxicity and genotoxicity. Following co-culture construction, cells were exposed to ENMs for 24 h. Following exposure, a complete media change was performed with media containing 3 µg/ml of cytochalasin B before incubating for 1.5 cell cycles (24 h). Cells were then removed with trypsin and fixed in 3% paraformaldehyde (PFA) for 24 h at 4 °C followed by permeabilization at 4 °C with 0.2% Triton X100. Cells were stained with 1 µg/ml anti-human CD-324 (FITC fluorophore) suspended in × 1 PBS and incubated at 4 °C in the dark for 45 min. Cells were subsequently washed in PBS before 100 µl of cells (per slide) were drop cast onto ethanol-cleaned glass slides which were left to air-dry and finally 30 µl of DAPI counterstain was applied. A total of 500BN TT1 cells were identified per replicate (1500BN total per concentration); TT1 cells were positively identified by their FITC fluorescence signal produced by e-cadherin, while d.THP-1 cells were selected against due to their absence of FITC fluorescence. Scoring was performed using the semi-automated Metafer microscope.

Since this is the first instance of TT1 cells being used with the in vitro micronucleus assay, the background levels of %Mn/BN had to be closely evaluated to determine if the cells were suitable for use with the assay. As the background level of Mn were less than 2% (specifically, 1.2%Mn/BN in the monoculture and 1.7%Mn/BN in the co-culture), the TT1 cells were deemed appropriate for use with the CBMN assay.

To investigate the potential role of oxidative stress, exposures were also performed with and without a two-hour incubation with 1.5 mM N-acetylcysteine (NAC). Following the NAC incubation, a complete media change was performed, and exposures were performed for 24 h. Cellular proliferation was evaluated due to the inclusion of cytochalasin B whereby the cytokinesis block proliferation index (CBPI) was calculated by Eq. (1) and % cytostasis by Eq. (2). The %cytostasis (an indicator of both cell viability and cell cycle stalling) was then used as a measure of %viable cells in the final graphs.

1 $${\text{CBPI}}\; = \;\left( {\text{Number\;of\;mononucleate\;cells}\; + \;\left( {2\; \times \;{\text{Number\;of\;binucleate\;cells}}} \right)\; + \;\left( {3X\;{\text{Number\;of\;multiucleate\;cells}}} \right)} \right)\;/{\text{Total\;number\;of\;cells}}$$

2 $$\% \;{\text{Cytostasis}}\; = \;100 - \;100\;\left( {\left( {{\text{CBPI}}_{T} \; - 1} \right)/\left( {{\text{CBPI}}_{C} \; - 1} \right)} \right)$$

where; T = ENM-treated cellsC = negative control.

### Data analysis and statistics

All data is presented as the mean ± the standard deviation (SD). Statistical analysis was performed in SPSS statistics software (v.20 IBM, UK) where all data sets were firstly analysed for normality (Shapiro–Wilk test, *p * ≤ 0.05) and for equal variance *p * ≤ 0.05)). Once all datasets were confirmed as possessing homogeneity of variance and were normally distributed a one-way analysis of variance (ANOVA) was performed with post hoc Dunnett’s multiple comparisons applied to evaluate pairwise statistical significance between control and concentrations; the alpha value was set to 0.05.

## Results

### FLG induces cytotoxicity and primary-indirect DNA damage in TT1 cells

**Fig. 1:**
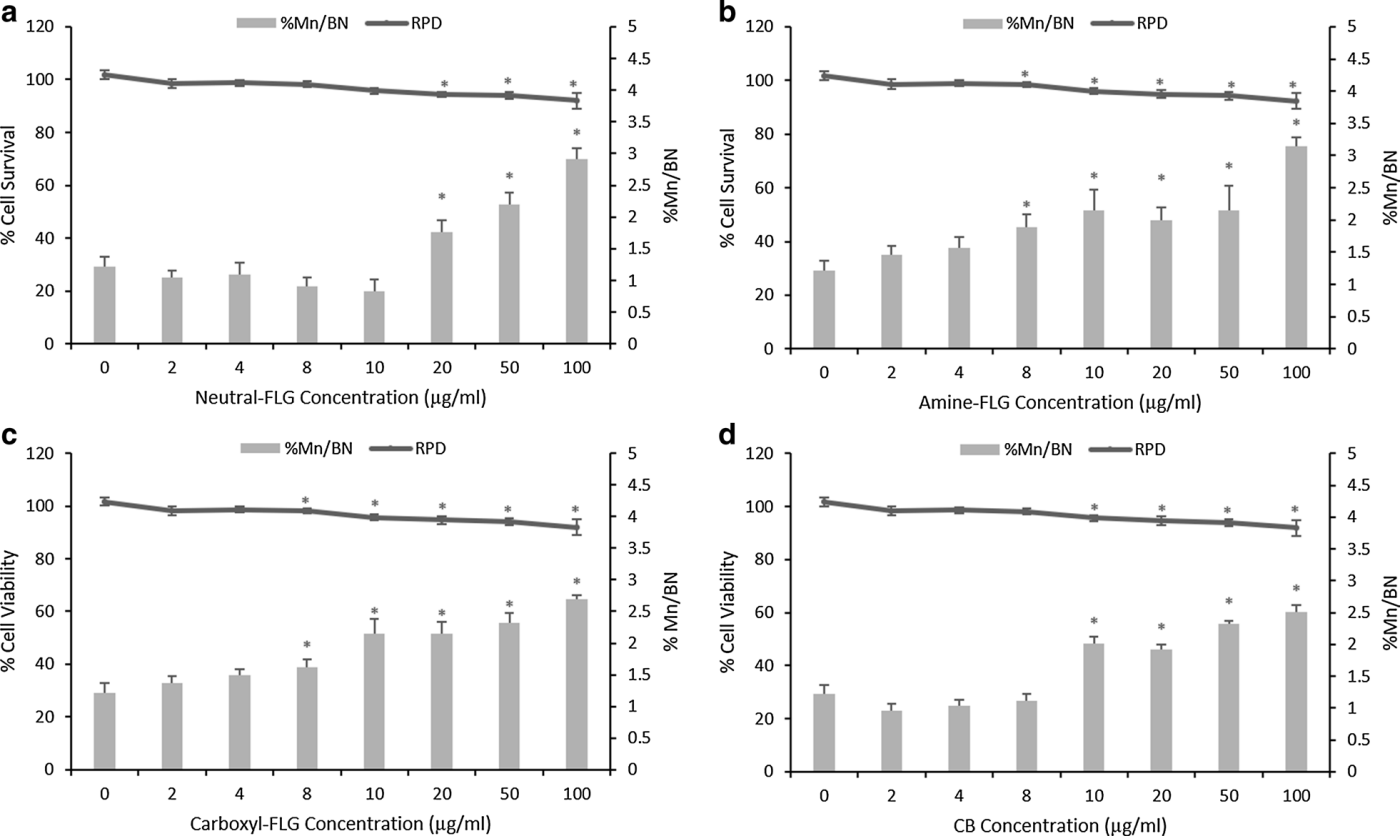
Cytotoxicity, evaluated through RPD and chromosomal damage assessment of TT1 cells utilising the in vitro CBMN assay following exposures to ENMs **a** neutral-, **b** amine-, **c** carboxyl-FLG and **d** CB particles. Results were considered significant (*) when *p* < 0.05. MMC (0.01 µg/ml) demonstrated a 6.2% Mn/BN frequency. Data is presented as the average value ± the standard deviation (SD), (*N* = 3)

This study aimed to evaluate the genotoxic potential of FLG and functionalised variants upon an epithelial barrier model of the lung. This was performed first in a monoculture of TT1 alveolar cells before constructing a co-culture model with d.THP-1 macrophages. Following a 24-h exposure, statistically significant cytotoxicity and genotoxicity were induced in TT1 cells revealing a potency trend of amine-FLG, carboxyl-FLG, CB, neutral-FLG. Whilst statistically significant (*p* < 0.05), this is unlikely to represent biological significance as viability did not drop below 80% for any tested ENM. Therefore, ENM cytotoxicity in TT1 cells are unlikely to be biologically detrimental given no appreciable cytotoxic levels were achieved. Genotoxicity however at the highest concentration of neutral-FLG and amine-FLG represented a onefold increase over baseline levels in monocultured TT1 cells at ~ 3.1% BN/Mn (Fig. **1**).

**Fig. 2:**
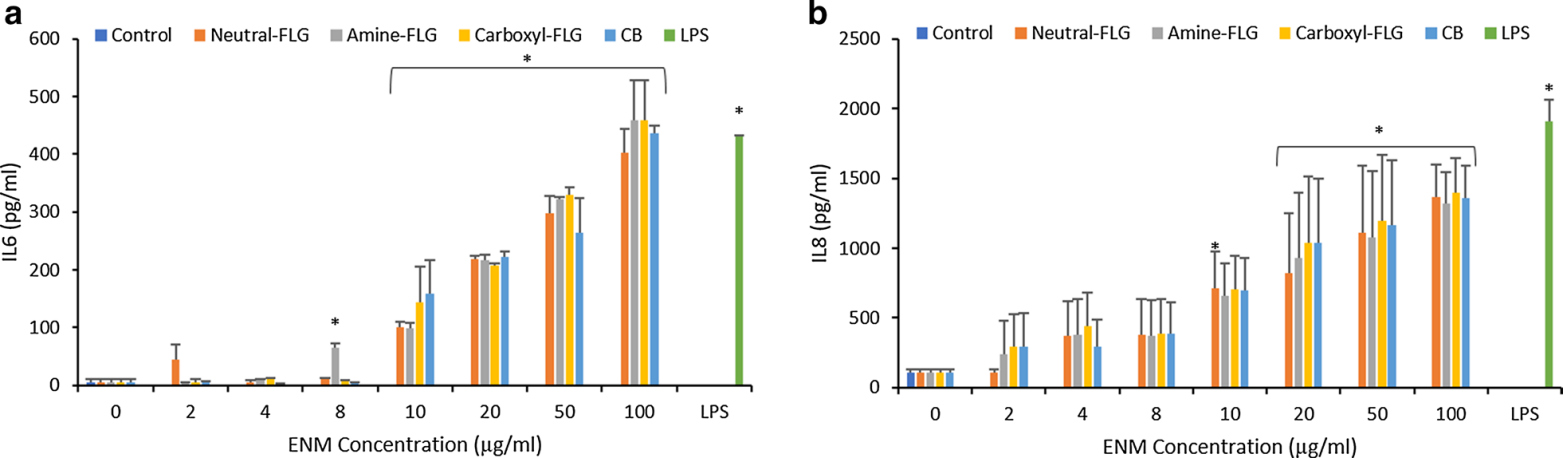
The quantification of (pro)-inflammatory cytokine IL-6 (**a**) and chemokine IL-8 (**b**) was performed following a 24-h exposure to test ENMs, LPS (100 ng/ml) served as the positive control. Cellular supernatant was harvested and stored appropriately until required. IL-6 showed low levels of activation at concentrations 2-8 µg/ml before a dose-dependent response was initiated with amine-FLG and carboxyl-FLG exceeding the levels reached by TT1 exposures to LPS. IL-8 levels were initially activated at 2-8 µg/ml before a dose dependent rise was observed becoming significant at 20 µg/ml whilst not exceeding LPS levels of activation. Results were considered significant (*) when *p* < 0.05. Data is presented as the average value ± the standard deviation (SD), (*N* = 3)

It was important to elucidate any key (pro)-inflammatory events in monocultured exposures prior to investigating secondary mechanisms in co-cultures. Therefore, IL-6 and IL-8, two key inflammatory mediators were quantified following 24-h exposures to both TT1 alveolar cells and d.THP-1 cells. TT1 IL-6 levels revealed a concentration-dependent increase, revealing an initial significant response at 8 µg/ml induced by amine-FLG at 66.2 pg/ml with a 12-fold increase over control levels (5.32 pg/ml). The concentration of IL-6 protein then steadily increased over the concentration range of 10-100 µg/ml with amine- and carboxyl-FLG the greatest response was at 100 µg/ml with levels of 458.3 and 458.7 pg/ml respectively, a 90-fold change. The initial significant IL-8 response was attributed to exposure with neutral-FLG at 10 µg/ml before each ENM elicited significant IL-8 at 20-100 µg/ml. At 20 µg/ml Neutral-, amine, Carboxyl-FLG and CB particles elicited an IL-8 protein concentration of 822.9, 930, 1038.5 & 1035.7 pg/ml respectively, demonstrating a 7-, 8-, 9- and ninefold increase over control IL-8 levels (106.34 pg/ml) respectively (Fig. 2).

**Fig. 3:**
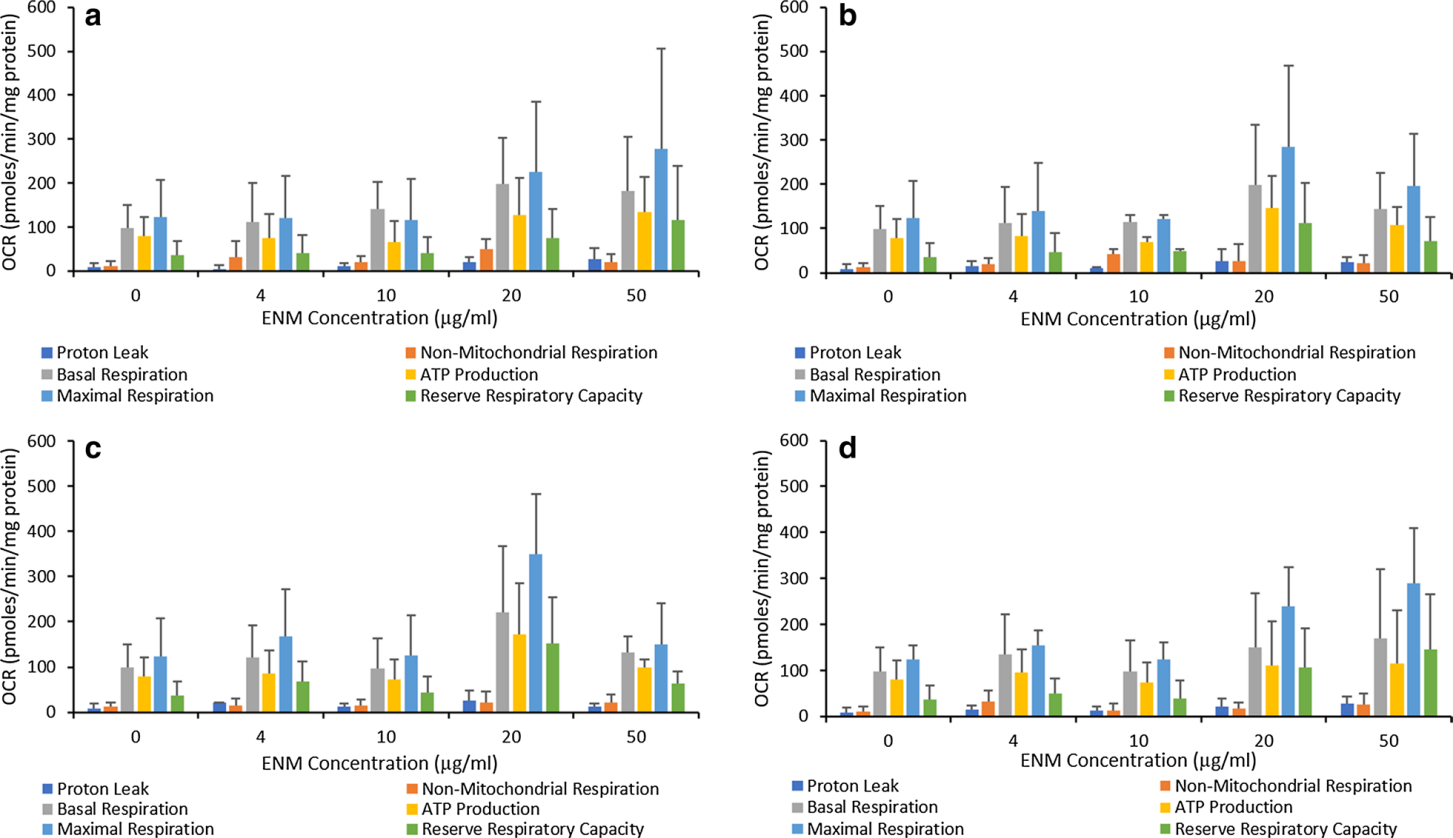
Mitochondrial metabolic parameters were calculated from oxygen consumption rates following 24-h exposures to neutral-FLG (**a**), amine-FLG (**b**), carboxyl-FLG (**c**) and CB (**d**). Mitochondrial activity was monitored using the Seahorse Bioanalyser with the TT1 cell setup in 24-well plates. Injection compounds and cellular seeding densities had been previously optimised for use with 24-well plates (data not shown). Results were considered significant (*) when *p* < 0.05. Data is presented as the average value ± the standard deviation (SD), (*N* = 3)

TT1 cells exposed to the ENMs showed a small, non-significant (*p* > 0.05) increase in mitochondrial function as the concentration of ENMs increased to 20 µg/ml for each ENM. Variation between metabolic features up to 10 µg/ml are difficult to interpret due to non-significant increases, which may be indicative of variation in the TT1 cells during the exposures. However, clear concentration-dependent increases can be seen when comparing basal and maximal respiration of control cells to levels following 20 µg/ml and 50 µg/ml exposures. At 20 µg/ml basal levels were raised two-fold when exposed to neutral-FLG, amine-FLG and carboxyl-FLG. Exposures at 50 µg/ml however revealed a depletion in basal respiration levels with respect to 20 µg/ml which may indicate agglomeration of ENMs preventing further elevation taking place. ATP production spiked at 20 µg/ml for all ENMs, carboxyl-FLG elicited the greatest response with a twofold increase. Maximal respiration in TT1 cells was raised at 20 µg/ml by each ENM, greatest with carboxyl-FLG at threefold over control levels. At 50 µg/ml, neutral-FLG and CB particles demonstrated a concentration-dependent increase by raising the reserve respiratory capacity by 40.3 and 38.8pmoles/min/mg protein respectively. Conversely, amine- and carboxyl-FLG depleted the reserve respiratory capacity by 40.9 and 87pmoles/min/mg protein with respect to data recorded at 20 µg/ml (Fig. 3).

### FLG induces significant cytotoxicity in d.THP-1 macrophages

**Fig. 4:**
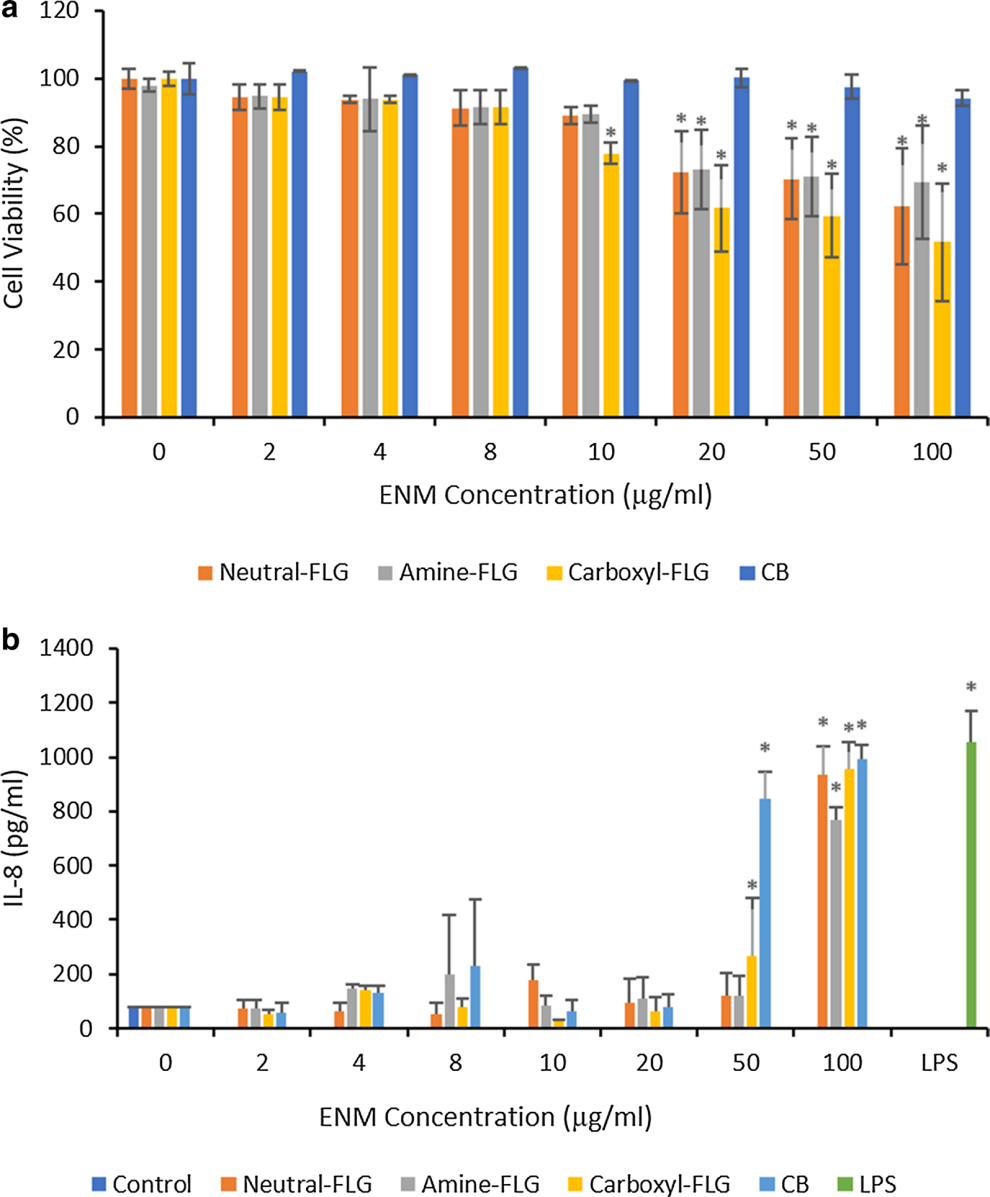
The cytotoxicity (**a**) of neutral-FLG, amine-FLG, carboxyl-FLG and CB upon differentiated THP-1 macrophages was determined by Trypan blue exclusion following a 24-h exposure. Supernatant was harvested and the (pro)-inflammatory response was quantified by analysing the release of IL-8 (**b**). THP-1 cells which had undergone differentiation following a PMA treatment at 50 nM for 24 h were then exposed to ENMs for a further 24 h. IL-8 levels in the d.THP-1 cells were quantified including an additional exposure with LPS (100 ng/ml) serving as a positive control. Results were considered significant (*) when *p* < 0.05. *N* = 3

Neutral-FLG exposure to d.THP-1 cells revealed a concentration-dependent decrease in viability up to 10 µg/ml, however concentrations of 20, 50 and 100 µg/ml promoted a 1.4, 1.4 and 1.6-fold decrease from control levels with cell viability falling to 72, 70 and 62%. This effect was also observed with exposures to amine-FLG however the final % viability here was 73, 71 and 64% at concentrations of 20, 50 and 100 µg/ml respectively. THP-1 cells exposed to carboxyl-FLG for 24-h showed greater sensitivity with the initial significant decrease in viability at 10 µg/ml whereby the cell viability was at 77%. A concentration-dependent trend was observed with viability recorded at 59 and 51% at 50 and 100 µg/ml, thus demonstrating the most potent effect on d.THP-1 viability with a twofold decrease at the highest exposure concentration. Lastly, d.THP-1 cells exposed to CB particles revealed no significant cytotoxicity however a small trend can be observed at 20, 50 and 100 µg/ml. The (pro)-inflammatory response of d.THP-1 cells was also performed following cytotoxicity testing focusing upon IL-8 due to the association of this chemokine with lung inflammation and IL-1β which had been highlighted in the literature. Analysis of IL-1β had been performed but no response was observed (data not shown). The d.THP-1 cells reached significant IL-8 levels at 100 µg/ml for each FLG material, while CB particles elicited significant IL-8 levels at 50 µg/ml (Fig. 4).

**Fig. 5:**
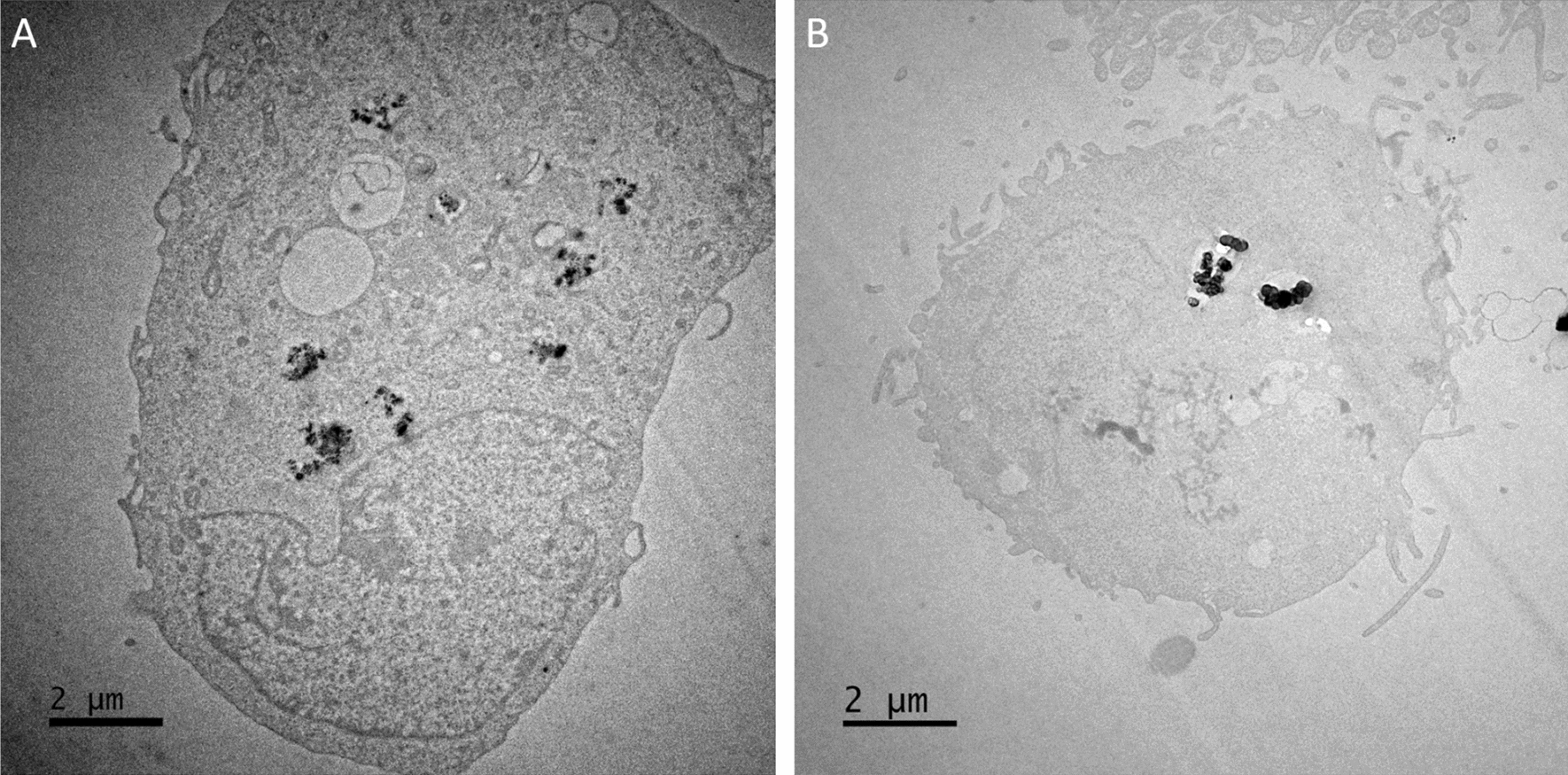
ENM uptake in monocultured TT1 cells exposed to **a** amine-FLG and **b** CB particles at 20 µg/ml was confirmed with TEM imaging. ENMs were observed within membrane-bound vesicles outside of the nucleus. Uptake was observed with all test ENM; surface charge (with respect to the graphene materials) did not appear to influence uptake. Quantitative analysis of uptake was not performed

Following a 24-h ENM exposure, TT1 cells were harvested, resin-embedded and sectioned for interaction and uptake confirmation. Electron micrographs of TT1 cells revealed each test ENM could be internalised by the epithelial cells. ENMs appeared to be contained within endocytic vesicles in almost all cases of uptake regardless of surface functionalisation, indicative of mediated endocytosis. Figure 5a reveals 1–2 agglomerates inside the cell without endosome wrapping, this could be the result of particles being released from a pre-existing endosome. There is also the possibility that these agglomerates do possess endocytic wrapping however the geometry of the particles imaged may be obscuring the vesicle. A 3-dimensional (3D) approach to TEM and sectioning could prove an ideal method of verifying this by capturing thicker cellular sections to construct a representative 3D image. Surface functionalisation appeared to be non-decisive in facilitating uptake as other publications have reported, whereby amine coatings (positively charged) can encourage cellular interaction. In the present study however, no quantitative analysis was performed on the number of particles internalised by the TT1 cells therefore we cannot definitively conclude that the amine groups increase uptake of amine-FLG particles over the other FLG materials used in this study. CB particles however did appear to be present in TT1 cells in lower concentrations than the FLG materials, it is unclear if this is a direct result of ENM morphology, however. Uptake was confirmed with each particle type however only representative images have been included of amine-FLG and CB particles. Confirmation of internalised FLG particles was achieved using fast Fourier Transform (FFT) and ImageJ software, as highlighted in the Additional file. The lattice spacing was confirmed to be 0.39 nm in the present study, confirmation of graphitic lattice spacing and not an amorphous carbon (Additional file 1: Fig. S1 and Fig. S2). Representative images of neutral-FLG and carboxyl-FLG uptake has been included in the (Additional file 1: Fig. S3).

### Detection of secondary mechanisms of DNA damage in TT1/d.THP-1 co-cultures

**Fig. 6:**
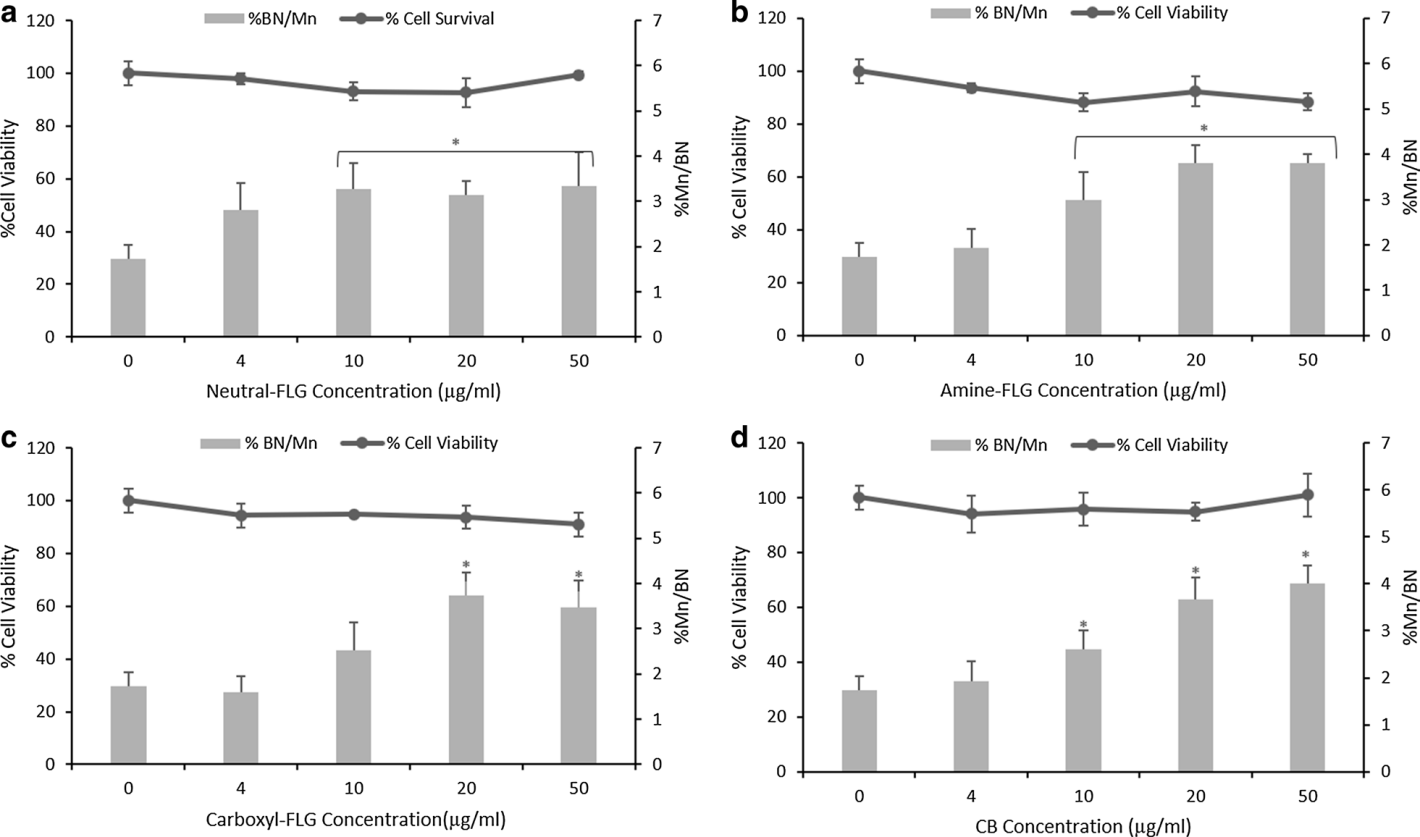
Co-culture cell viability and chromosomal damage was evaluated by CBPI and the in vitro CBMN assay. Co-culture models exposed to neutral-FLG (**a**), amine-FLG (**b**), carboxyl-FLG (**c**) and CB (**d**) showed no significant cytotoxicity, each ENM induced a genotoxic response at concentrations of 20-50 µg/ml with only carboxyl-FLG not causing genotoxicity at 10 µg/ml. Results were considered significant (*) when *p* < 0.05. MMC (0.01 µg/ml) demonstrated a 7.2% Mn/BN frequency. Data is presented as the average value ± the standard deviation (SD), (*N* = 3)

**Fig. 7:**
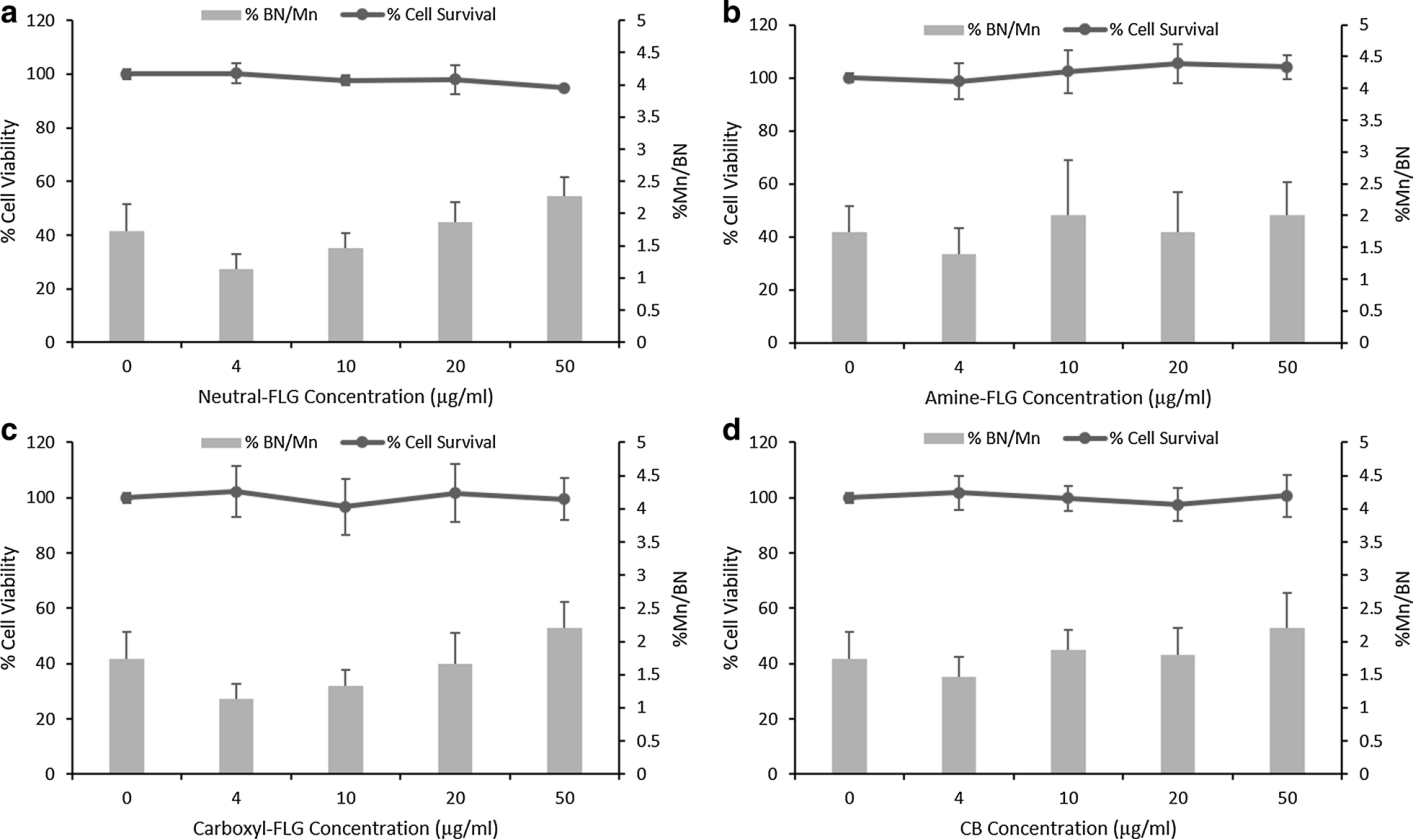
The effect of NAC pre-incubation on co-culture cell viability and chromosomal damage was evaluated by CBPI and the in vitro CBMN assay. Co-culture models exposed to neutral-FLG (**a**), amine-FLG (**b**), carboxyl-FLG (**c**) and CB (**d**) showed no significant cytotoxicity, moreover the presence of 1.5 mM NAC prior to exposures appeared to reduce the genotoxic impact of each test ENM to where no significant response was observed. Results were considered significant (*) when *p* < 0.05. MMC (0.01 µg/ml) demonstrated a 6.2% Mn/BN frequency. Data is presented as the average value ± the standard deviation (SD), (*N* = 3)

Following the monoculture exposures, TT1 alveolar cells and d.THP-1 cells were co-cultured together to determine whether secondary mechanisms of genotoxicity could be observed. Cell proliferation and DNA damage in the co-culture was assessed by the in vitro CBMN assay adapted for use with co-cultures as detailed by Evans and colleagues [9]. No significant cytotoxic responses were observed in co-cultures however significant DNA damage was observed with the lowest observed genotoxic effect level (LOGEL) was 10 µg/ml for neutral-FLG, amine-FLG and CB particles whilst the LOGEL for carboxyl-FLG was observed at 20 µg/ml. At 50 µg/ml TT1 cell %BN/Mn reached a ~ twofold increase over control levels (Fig. 6). Given there was a ~ twofold increase in concentrations of ENMs in co-culture exposures compared to monocultured TT1 exposures, secondary mechanisms were hypothesised to be present. To investigate these effects, co-culture exposures were repeated following a two-hour NAC (1.5 mM) exposure which would theoretically provide a means for oxidative radicals to be scavenged before they could cause DNA damage. Co-culture ENM exposure resulted in no significant changes in cell viability or DNA damage as compared to the untreated control. Crucially, at the highest test concentration of 50 µg/ml the %BN/Mn had decreased from an average 3.65% in experiments without NAC to 2.18% in co-cultures which had the NAC pre-treatment (Fig. 7). This result indicates that the genotoxicity observed was, if at least in part, mediated by oxidative stress when considering both FLG and CB in the co-culture. The NAC treatment appeared therefore to scavenge oxidative products produced by the d.THP-1 macrophages before they could damage surrounding epithelial TT1 cells. A summary of these results is provided in Table 1.

**Table 1:** Summary of conclusions when comparing data generated in in vitro monocultures and co-culture models

	Cytotoxicity	Genotoxicity	(Pro)-inflammatory response	Mitochondrial stress	Uptake
Monocultured TT1	Significant (*p* < 0.05) effect observed with all ENMs, earliest significant response observed at 8 µg/ml (Amine-FLG and carboxyl-FLG, CB, neutral-FLG)	Significant (*p* < 0.05) effect observed with all ENMs, earliest significant response observed at 8 µg/ml (Amine-FLG, carboxyl-FLG, CB, neutral-FLG)	Significant (*p* < 0.05) effect observed with IL-6 & IL-8 with all ENMs, earliest significant response observed at 8 µg/ml (IL-6), 10 µg/ml (IL-8)	Non-significant (*p* > 0.05) effect observed	Uptake observed with each test ENM
Monocultured d.THP-1	Significant (*p* < 0.05) effect observed with FLG materials, earliest significant response observed at 10 µg/ml (Carboxyl-FLG, neutral-FLG, amine-FLG)	N/A	Significant (*p* < 0.05) effect observed with IL-8 with all ENMs, earliest significant response observed at 50 µg/ml	N/A	N/A
Co-cultured TT1/d.THP-1	Non-significant (*p* > 0.05) effect observed	Significant (*p* < 0.05) effect observed without NAC with all ENMs, earliest significant response observed at 10 µg/ml Non-significant (*p* > 0.05) effect observed with NAC with all ENMs	N/A	N/A	N/A

A hazard ranking appears where direct comparisons were possible to differentiate between the test ENMs. Not applicable (N/A) appears in the table where a test was not performed or could not be optimised in the timeframe. The earliest significant response concentration is given per category

## Discussion

This study aimed to evaluate the scope of genotoxic mechanisms induced by FLG with varying surface chemistries within an advanced co-culture of TT1/d.THP1 cells. It is crucial to understand the fundamental hazard posed by FLG materials given their tremendous potential in reinforced nanocomposites which have large-scale consumer demand. As previously stated, studies focused upon specifically FLG, exposures to lung cells are sparse in the literature and furthermore the underlying mechanisms can be overlooked. Whilst numerous studies have been performed using graphene-based nanoplatelets, nanosheets or GO in lung models, making direct comparisons with FLG becomes problematic due to the nomenclature of this specific ENM. It was imperative that the present study seek to understand how to distinguish primary and secondary mechanisms of genotoxicity using the relevant models. Secondly, to incorporate an understanding of the physico-chemical features of the materials and how they relate to the observed biological responses in each endpoint. This investigation will (to the authors knowledge) be the first to evaluate FLG impact upon an advanced alveolar barrier model, thus providing a foundation for further research attempting to elucidate mechanisms of toxicity in similar models.

Utilising monocultures of alveolar TT1 cells and d.THP-1 macrophages, this study has demonstrated the capacity for FLG materials to induce (pro)-inflammatory effects and genotoxicity. ENMs have been reported to induce genotoxic and (pro)-inflammatory effects in monocultured human bronchial epithelial (16HBE14o^−^) cells and immune cells. For instance, in the work by Evans and colleagues who used two types of superparamagnetic iron oxide nanoparticles (SPIONs) only the Fe_2_O_3_ was genotoxic in monocultured 16HBE14o^−^ cells. The other particle type, Fe_3_O_4_ was not capable of promoting genotoxicity; however both particle types elicited a strong tumour necrosis factor (TNF-α) and IL-8 response over a similar concentration range as the present study [9]. Most recently, in the study by Burgum and colleagues the cytotoxic and genotoxic effect of the ENMs used in the present study were evaluated in monocultured 16HBE14o^−^ cells over the same concentration range. The authors reported no cytotoxic effects with neutral-FLG and amine-FLG inducing significant DNA damage at the higher concentrations [1]. Whilst studies investigating the genotoxicity of FLG on lung cells are few, CB particle exposure is much more common. In vivo CB exposure causes malignant tumours in test animals possibly due to chronic inflammation of the lungs which could lead to carcinogenesis [13]. To understand the mechanistic impact of CB particles, Hiraku and colleagues exposed 56 nm (CB56) and 95 nm (CB95) diameter CB particles to A549 lung epithelial cells and RAW 264.7 macrophages at 0-50 µg/ml. Both particle types induced the formation of 8-nitroguanine, a nitrative DNA legion. Further oxidative damage was elucidated in the study whereby CB95 induced greater ROS in macrophages as well as producing greater amounts of nitric oxide (NO) than did CB65. Hiraku and colleagues also demonstrated uptake in their study was through clathrin-mediated endocytosis [13].

IL-6 and IL-8 were both significantly elevated in the present study which is consistent with the observed genotoxicity in TT1 cells and supported by TEM-confirmation of uptake. TT1 cells have been reported capably internalising functionalised multi-walled carbon nanotubes (f-MWCNTs) however no information currently exists on their interaction with graphene [23]. Exposures to TT1 cells revealed no significant effect on the mitochondrial function in the present study, similar work has been reported in the literature by Jantzen and colleagues who exposed diesel exhaust particles (DEP) up to 100 µg/ml to THP-1a cells. Unlike the present study, Jantzen et al. attempted the same procedure with A549 cells however the cells OCR rate proved too low, THP-1a cells did respond with significant elevations in basal respiration, however. The authors also noted respiratory capacity was significantly elevated following exposures at concentrations of 25 and 100 µg/ml [14]. Similarly, Burgum et al. performed (pro)-inflammatory and mitochondrial analysis of FLG upon monocultured 16HBE14o^−^ cells and observed significant elevation in IL-8 levels and significant depletion in mitochondrial function including ATP production. These effects were largely dependent upon surface characterisation where carboxyl groups appeared to hinder ATP production more than amine groups [1].

Graphene-associated toxicity in d.THP-1 macrophages has been demonstrated by Li and colleagues, whereby hydrated GO (hGO) demonstrated the strongest cytotoxic effect in macrophages. The authors noted the presence of carbon radicals (introduced in the hydration of GO) caused lipid peroxidation of the cell membrane, membrane lysis and ultimately cell death [17]. The lack of cytotoxicity from CB particles in d.THP-1 cells can be supported by findings in the literature whereby 100 nm CB particle exposures to A549 and THP-1 cells at 16 µg/ml showed no cytotoxic effects in the Alamar Blue assay [6]. More recently, Gurunathan and colleagues were able to demonstrate immunotoxicity of GO and vanillin-functionalised GO (V-rGO) on THP-1 cells with further notable effects of loss in cell viability, proliferation in a dose-dependent manner, increased lactate dehydrogenase levels, ROS production and depletion of mitochondrial membrane potential [12]. Seemingly providing evidence for similar modes of action to the present study, with emphasis on the carboxyl-FLG which had the strongest cytotoxic effect of each ENM on d.THP-1 cells and bearing the closest resemblance with chemical reactivity to the V-rGO. Juan Ma and colleagues in 2015 reported size-dependent differences of GO inducing both mouse macrophage and human THP-1 activation. The authors reported large GO of 1300 nm lateral diameter at 20 µg/ml could induce greater TLR activation which contributed to a more potent NF-kB pathway induction. Juan Ma et al. were then able to detect significant levels of TNF-α, IL-6 and IL-1β in the media [19]. The link between IL-1β secretion and graphene exposures has been reported in the literature. Schinwald and colleagues demonstrated graphene nanoplatelets could activate the inflammasome complex and detected significant IL-1β following 24 h THP-1 exposures at 1, 5 and 10 µg/cm^2^ [24]. The present study did not detect any response from d.THP-1 cells with regards to IL-1β at the tested concentrations for each material (data not shown). This may suggest an alternative signalling pathway being activated in d.THP-1 cells independent of the inflammasome complex, this will almost certainly be specific to the types of ENMs under study.

The co-culture model of TT1/d.THP-1 cells was constructed to better mimic the architecture of the alveolar region of the lung using a dual-cell model. Genotoxicity induced in the co-culture model occurred at lower concentrations and represented a greater %BN/Mn frequency, than levels reached in monoculture exposures of TT1 cells and in 16HBE14o^−^ cells, tested in a separate investigation [1]. This effect was demonstrated by Evans et al. whereby secondary mechanisms of genotoxicity elevated the frequency of DNA damage across the concentration range following dextran-coated superparamagnetic iron oxide nanoparticle (dSPION) exposures [9]. NAC treatment for 2 h prior to exposures in the present study eliminated the genotoxicity within co-cultures at all concentrations for each test FLG. This major finding supports the theory that secondary mediators such as NO were readily scavenged by excess antioxidant levels in the culture conditions.

In the present study, it was hypothesised that in the co-culture exposures the d.THP-1 macrophages would phagocytose the ENMs, which in turn would result in superoxide generation and depletion of mitochondrial function. The oversaturation of the mitochondrial enzyme manganese superoxide dismutase (Mn-SOD) could then activate nicotinamide adenine dinucleotide phosphate (NADPH), dislocating cytochrome c and inducing subsequent apoptosis of d.THP-1 macrophages. An increase in the expression of p53, NF-kβ and NOX2 could then be tested for. The release of (pro)-inflammatory mediators and nitric oxide NO into the co-culture environment would then exert lipid peroxidation on the TT1 cells. This provides a clearly testable hypothesis as NO can be converted in to stable products; nitrite and nitrate (NO_2_^−^ and NO_3_^−^) which can then be quantified using fluorescent techniques [3]. It is known that whilst NAC boost intracellular glutathione levels, it also has reducing potential through its thiol-disulphide exchange activity. NAC may also possess other beneficial processes such as inhibiting p38 MAP kinase, activating protein-1 as well as NF-kB transcription factors [28], all of which would flood the cells in a chronic (pro-inflammatory response upon ENM exposure over 24 h.

Fukai and colleagues investigated the genotoxic effects of multi-walled carbon nanotubes (MWCNTs) on murine lung resident (GDL1) cells and RAW 264.7 macrophages. The authors reported a stronger mutation frequency in co-cultured GDL1 cells and RAW 264.7 macrophages over monocultured GDL1 cells noting a greater IL-1α and IL-1β production when macrophages were present. The authors also reported the presence of the free-radical-induced DNA lesion, 8-hydroxydeoxyguanosine (8-OHdG), in co-cultured GLD1 cells was greater than monocultured cells indicative of secondary mechanisms [11]. In the recent work of Evans and colleagues investigating SPION toxicity in co-cultures of 16HBE14o^−^ and d.THP-1 macrophages, the authors reported secondary genotoxicity at concentrations of 10, 50 and 100 µg/ml. This was represented by a rise in the %BN/Mn over monocultured 16HBE14o^−^ cells [9]. In the latter study, the authors also demonstrated particle uptake in both cell types with NPs contained within endocytic vesicles indicative of endocytosis rather than passive uptake. However, diesel exhaust particles (DEPs) used in the study by Jantzen and colleagues revealed greater genotoxicity in monocultured A549 cells rather than a co-culture of A549 cells grown with THP-1a cells [14]. The current battery of tests for in vitro nano-safety primarily focus on monocultured cells whereas this study and others before clearly demonstrated the value of combining multiple cell types to explore secondary mechanisms of genotoxicity. Whereas the THP-1a cells provided a protective role in the work by Jantzen et al., in the present study the d.THP-1 cells appear to have enhanced the genotoxic effects of FLG and CB. This demonstrates the need for more in vitro nanosafety studies utilising advanced models to distinguish the mechanistic differences observed in the literature thus far.

## Conclusions

Neutral-, amine-, carboxyl-FLG & CB particles each induced significant primary-indirect genotoxicity in monocultured TT1 cells. Furthermore, significant cytotoxicity was observed in d.THP-1 macrophages following a 24-h exposure period. The ENMs were able to gain entry to the TT1 monocultured cells (evidenced by TEM) and were observed within membrane-bound vesicles. Surface functionalisation appeared to play no role in preferential uptake of FLG materials. Each test ENM promoted significant genotoxicity via secondary mechanisms in co-cultured TT1/d.THP-1 cells. Co-culture genotoxicity was lowered to non-significant levels when the model was pre-exposed to 1.5 mM of NAC for two hours prior to exposures. This observation is evidence to support the hypothesis that oxidative stress is the driving mechanism behind the genotoxicity induced by the FLG and CB following exposure to an alveolar co-culture barrier model.

## Supplementary Information

**Additional file 1: Fig. S1.**Methodology for determining the interlayer spacing of graphene particles in TT1 cells. Figure S2. Fast Fourier Transform (FFT) analysis of internalised FLG. FFT provides structural information regarding the electronic arrangement of the crystal. Figure S3. Neutral-FLG (**a**) and carboxyl-FLG (**b**) exposure to TT1 cell at 20µg/ml.

## Data Availability

The data sets used/analysed in this manuscript are available from the corresponding author under reasonable request.
