# Supplementary material for: Few-layer graphene induces both primary and secondary genotoxicity in epithelial barrier models in vitro
Source: J Nanobiotechnology. 2021 Jan 19;19:24. doi: 10.1186/s12951-021-00769-9 (PMC7816456; doi:10.1186/s12951-021-00769-9)
Supplement: Supplementary file 1 — Additional file 1: Fig. S1.Methodology for determining the interlayer spacing of graphene particles in TT1 cells. Figure S2. Fast Fourier Transform (FFT) analysis of internalised FLG. FFT provides structural information regarding the electronic arrangement of the crystal. Figure S3. Neutral-FLG (a) and carboxyl-FLG (b) exposure to TT1 cell at 20µg/ml. [file 12951_2021_769_MOESM1_ESM.docx]

**TITLE: Few-layer graphene induces both primary and secondary genotoxicity in epithelial barrier models *in vitro***

Michael J Burgum^1^, Martin JD Clift^1^, Stephen J Evans^1^, Nicole Hondow^2^, Afshin Tarat^3^, Gareth J Jenkins^1^ and Shareen H Doak*^1^

1. Institute of Life Science, Swansea University Medical School, Swansea University, Singleton Park, Swansea SA2 8PP, Wales, UK.

2. School of Chemical and Process Engineering, University of Leeds, Leeds, LS2 9JT, UK.

3. Perpetuus Carbon Technologies, Unit B1, Olympus Court, Millstream Way, Swansea Vale, Llansamlet, SA70AQ.

*Corresponding Author:

# Tel: +44 1792 295388

Fax: +44 1792 602147

E-mail address: s.h.doak@swansea.ac.uk


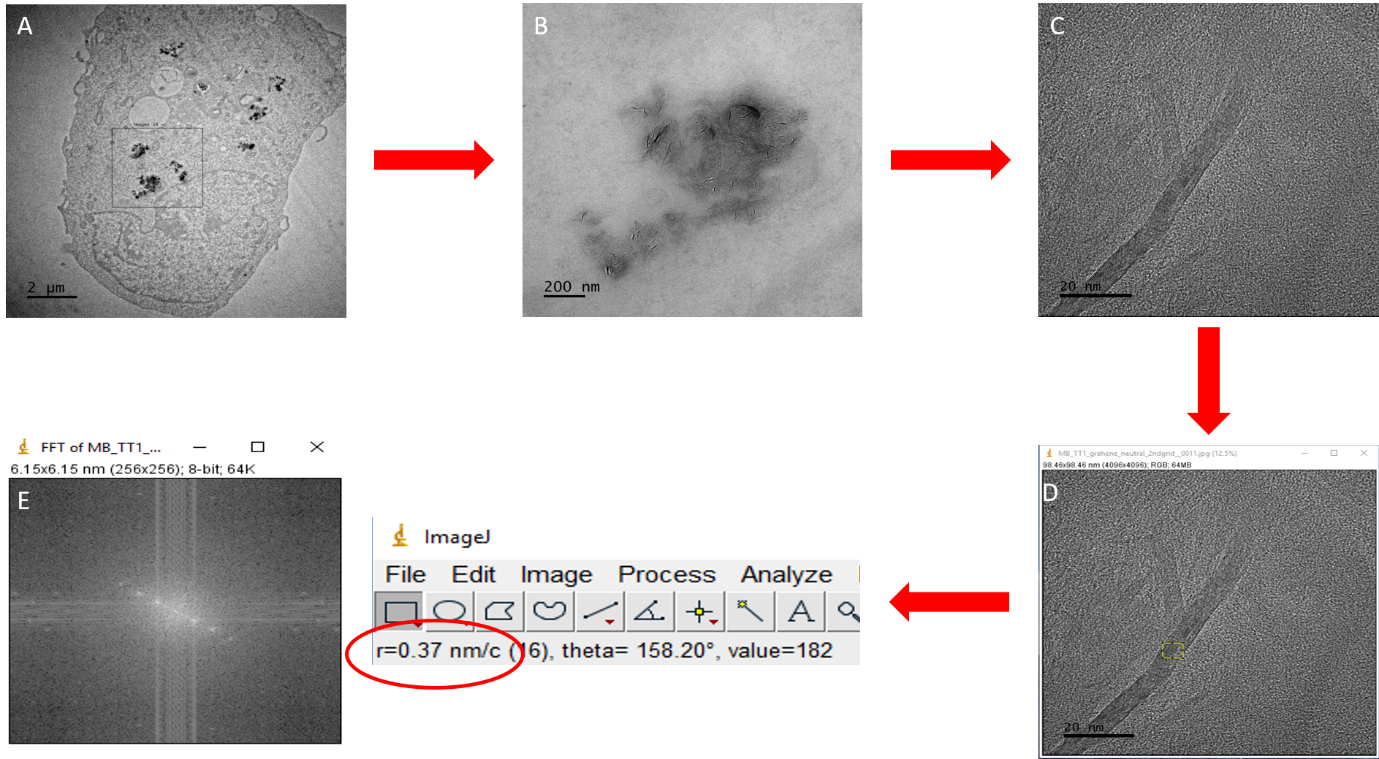


**Additional Figure 1.** Methodology for determining the interlayer spacing of graphene particles in TT1 cells. Firstly, a TT1 cell was located containing ENMs (A), then high resolution focusing is performed to visualise the FLG contained within an endocytic vesicle (B). A FLG crystal is then focused upon in (C & D) whereby the FLG stack is oriented on its Z-axis. Once the image has been captured (E), fast Fourier transform (FFT) can be performed using ImageJ to provide the final output.


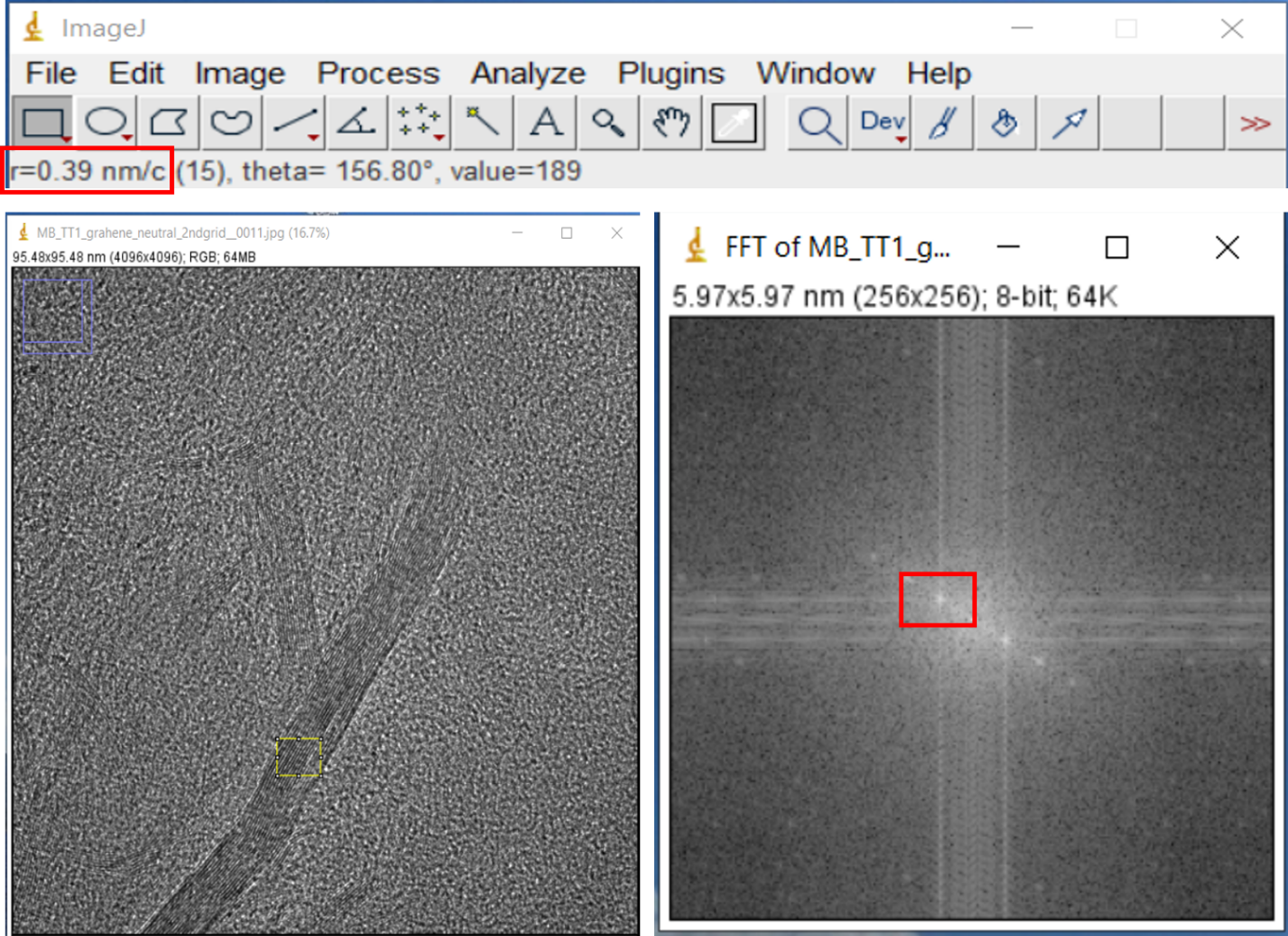


**Additional Figure 2.** Fast Fourier Transform (FFT) analysis of internalised FLG. FFT provides structural information regarding the electronic arrangement of the crystal. Electron density will always be strongest at the atomic position which then allows the user to measure the spacing between the layers of graphene which in the case of this Neutral-FLG sample is 0.39nm, exactly what you would expect the atomic spacing of graphitic material to be. Analysis of this type typically allows a (+/-) 10% in standard deviation to be identifiable as sp^2^ hybridised graphitic crystal by nature.

1.
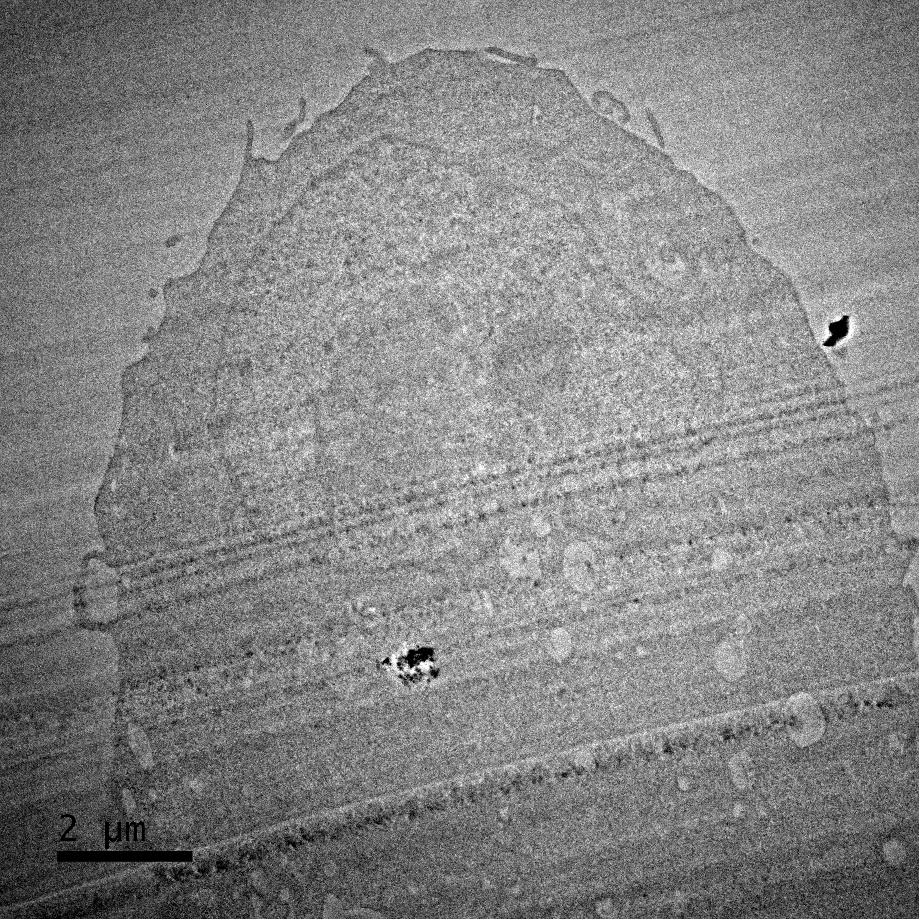

2.
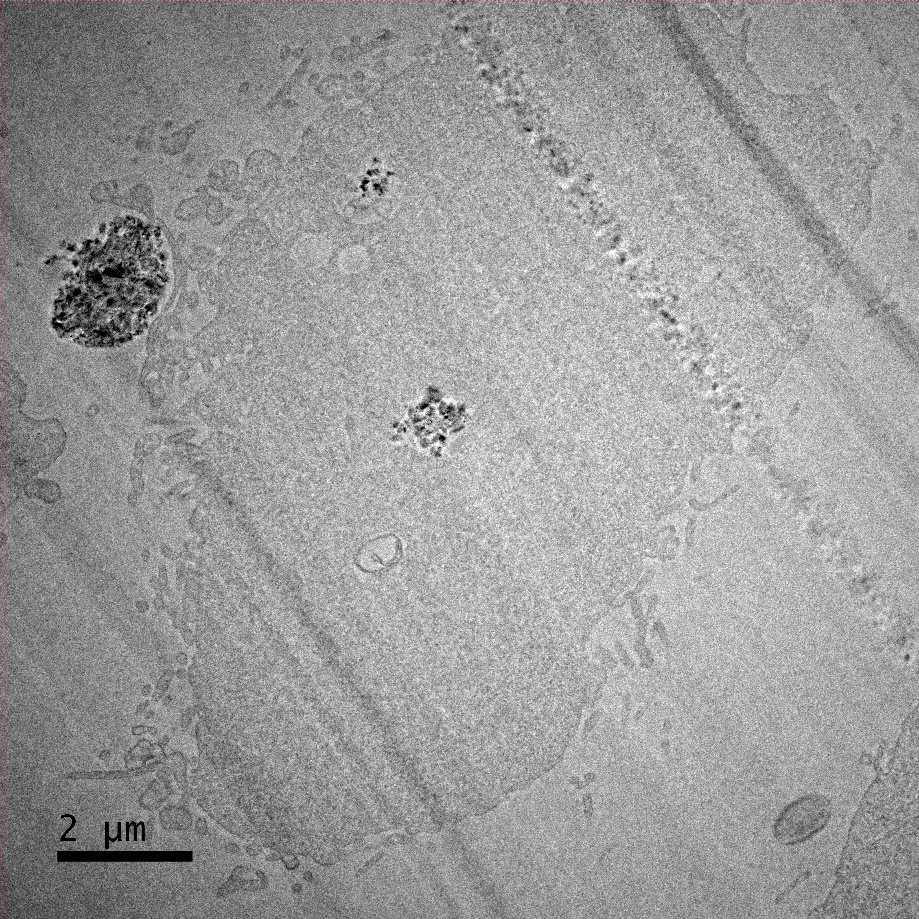


**Additional Figure 3.** Neutral-FLG (A) and carboxyl-FLG (B) exposure to TT1 cell at 20µg/ml. Damage from sectioning can be seen across the images, however both particle types are observed within TT1 cells, within endocytic vesicles.
